# Ginkgo biloba extract attenuates the disruption of pro-and anti-inflammatory T-cell balance in peripheral blood of arsenicosis patients

**DOI:** 10.7150/ijbs.39351

**Published:** 2020-01-01

**Authors:** Shiqing Xia, Qian Sun, Zhonglan Zou, Yonglian Liu, Xiaolin Fang, Baofei Sun, Shaofeng Wei, Dapeng Wang, Aihua Zhang, Qizhan Liu

**Affiliations:** 1 The Key Laboratory of Environmental Pollution Monitoring and Disease Control, Ministry of Education, Department of Toxicology, School of Public Health, Guizhou Medical University, Guiyang 550025, Guizhou, People's Republic of China.; 2 Center for Global Health, China International Cooperation Center for Environment and Human Health, School of Public Health, Nanjing Medical University, Nanjing 211166, Jiangsu, People's Republic of China.; 3 The Key Laboratory of Modern Toxicology, Ministry of Education, School of Public Health, Nanjing Medical University, Nanjing 211166, Jiangsu, People's Republic of China.

**Keywords:** Arsenicosis, Ginkgo* biloba* extract, T helper 17 cells, Regulatory T cells.

## Abstract

Endemic arsenicosis is a public health problem that affects thousands of people worldwide. However, the biological mechanism involved is not well characterized, and there is no specific treatment. Exposure to arsenic may be associated with immune-related problems. In the present work, we performed an investigation to determine whether the Th17/Treg balance was abnormal in peripheral blood mononuclear cells (PBMCs) of patients with arsenicosis caused by burning coal. Furthermore, we investigated the effect of *Ginkgo biloba* extract (GBE) on the Th17/Treg imbalance in patients with arsenicosis. In this trial, 81 arsenicosis patients and 37 controls were enrolled. The numbers of Th17 and Treg cells, as well as related transcription factors and serum cytokines, were determined at the beginning and end of the study. Patients with arsenicosis exhibited higher levels of Th17 cells, Th17-related cytokines (IL-17A and IL-6), and the transcription factor RORγt. There were lower levels of Treg cells, a Treg-related cytokine (IL-10), and the transcription factor Foxp3 as compared with controls. There was a positive correlation between the levels of Th17 cells and IL-17A and the levels of arsenic in hair. Arsenicosis patients were randomly assigned to a GBE treatment group or a placebo group. After 3 months of follow-up, 74 patients completed the study (39 cases in the GBE group and 35 in the placebo group). Administration of GBE to patient upregulated the numbers of Treg cells and the level of IL-10 and downregulated the numbers of Th17 cells and the levels of cytokines associated with Th17 cells. The mRNA levels of Foxp3 and RORγt were increased and decreased, respectively. These results indicated that exposure to arsenic is associated with immune-related problems. The present investigation describes a previously unknown mechanism showing that an imbalance of pro- and anti-inflammatory T cells is involved in the pathogenesis of arsenicosis and that a GBE exerts effects on arsenicosis through regulation of the pro- and anti-inflammatory T cell balance.

## Introduction

Endemic arsenicosis is a worldwide disease that causes numerous pathological effects 1. In the Guizhou province of China, arsenic poisoning caused by the burning of coal in unventilated indoor stoves is a common means of exposure, and more than 200,000 villagers are at risk for such exposures 2. Epidemiological evidence reveals that chronic exposure to derivatives of arsenic, a metalloid, is associated with skin, lung, and liver cancer and with various non-cancerous disorders, including atherosclerosis, hypertension, cerebrovascular disease, and diabetes 3, 4. A variety of mechanisms, such as oxidative stress, DNA damage, disrupted signal transduction, and epigenetic changes, have been proposed for the arsenic-induced deleterious health effects 5, 6. An immune dysfunction may also be involved 7.

As components of the adaptive immune system, naïve CD4^+^ T cells, following activation by antigen presentation, differentiate into specific effector T-helper (Th) cell subsets, which regulate immunity and inflammation 8. Adaptive immunity mediated by T cells is involved in the pathogenesis and progression of arsenic poisoning, which can lead to increased risk of infections and chronic diseases, including cancers 9. Although T helper 17 (Th17) and T regulatory (Treg) cells both differentiate from naïve CD4^+^ T cells, they are distinct populations that have functionally opposite effects 10. Th17 cells represent a pro-inflammatory subset that expresses retinoic acid-related orphan receptor **γ**t (RORγt) and is involved in the development of autoimmunity and allergic reactions by producing IL-17 11, 12; Treg cells express the forkhead/winged helix transcription factor 3 (Foxp3), have an anti-inflammatory role, and maintain tolerance to self-components through direct contact with cells or by releasing anti-inflammatory cytokines, such as IL-10 and transforming growth factor-β1 (TGF-β1) 13, 14. The ratio of Th17/Treg cells regulates immune activities, and Th17/Treg imbalances may provide a basis for understanding the immunological mechanisms that induce and regulate autoimmunity and chronic inflammation 15. Acute oral administration of arsenite, an activated form of arsenic, to experimental animals disturbs the immune homeostasis in lung tissue, with lessened Th17 differentiation and elevated Treg differentiation 16. Further, acute exposure to arsenite leads to an imbalance between Th17 and Treg cells in the spleen and thymus of C57BL/6 mice 17. Low concentrations of arsenite inhibit the expression and secretion of IL-17A by human naïve and memory Th17 cells 18. Arsenite exposure affects T cell secretion of cytokines by up-regulating the Treg population and thereby causing immune inhibition 19. Although the links between exposure to inorganic arsenic and Th17/Treg immune dysfunction have been described to some extent, there has been inadequate characterization of the effects of arsenic on the Th17/Treg immune balance in human populations suffering from arsenic poisoning caused by coal-burning. Moreover, little is known about the immunological changes caused by arsenic in human Th17 and Treg cells.

*Ginkgo biloba* (GB) has been used in traditional Chinese medicine for several hundred years. *Ginkgo biloba* extract (GBE), obtained from GB leaves, contains ginkgo flavone glycosides, terpene lactones, and other active components. It has been used to treat various diseases, including cardiovascular and neurological disorders 20-22. Various mechanisms have been proposed for the beneficial effects of GBE, including antioxidant functions, anti-inflammatory effects, inhibition of platelet aggregation, and immune regulation 23-26. Dietary supplementation with GBE enhances the immune organ index and the number of T lymphocytes and adjusts the balance of T lymphocyte subsets, thereby enhancing immune function 27, 28. In rats with arsenic poisoning caused by coal-burning, *Ginkgo biloba* diminishes kidney damage and improves immune function 29. Nevertheless, the specific role and mechanism of GBE against immune damage caused by arsenic are poorly understood, especially for populations exposed to arsenic.

Our previous studies of arsenic-poisoned populations in Guizhou province found that arsenic from coal fires induces multiorgan damage. The prevalence of skin, liver, and kidney damage in the arsenic-exposed area is higher compared with the reference group (χ2 = 184.42, 24.16, 8.25; *P* all < 0.01) 30. In the present investigation, we first determined whether the Th17/Treg (pro- and anti-inflammatory T cell) balance was disturbed in subjects chronically exposed to arsenic and explored the potential role of atopy in arsenic poisoning. With a population exposed to arsenic by coal burning, we determined the numbers of Th17 and Treg cells in PBMCs, the relative levels of transcription factors (RORγt and Foxp3), and serum levels of Th17- and Treg-related cytokines. In addition, for this population, we examined the regulatory effects of GBE on the Th17/Treg response. Our results provide data relating to a mechanism and to a possible drug intervention for preventing and/or reversing immune damage due to arsenic poisoning caused by coal burning.

## Results

### Demographic data of the subjects

**Figure 1:**
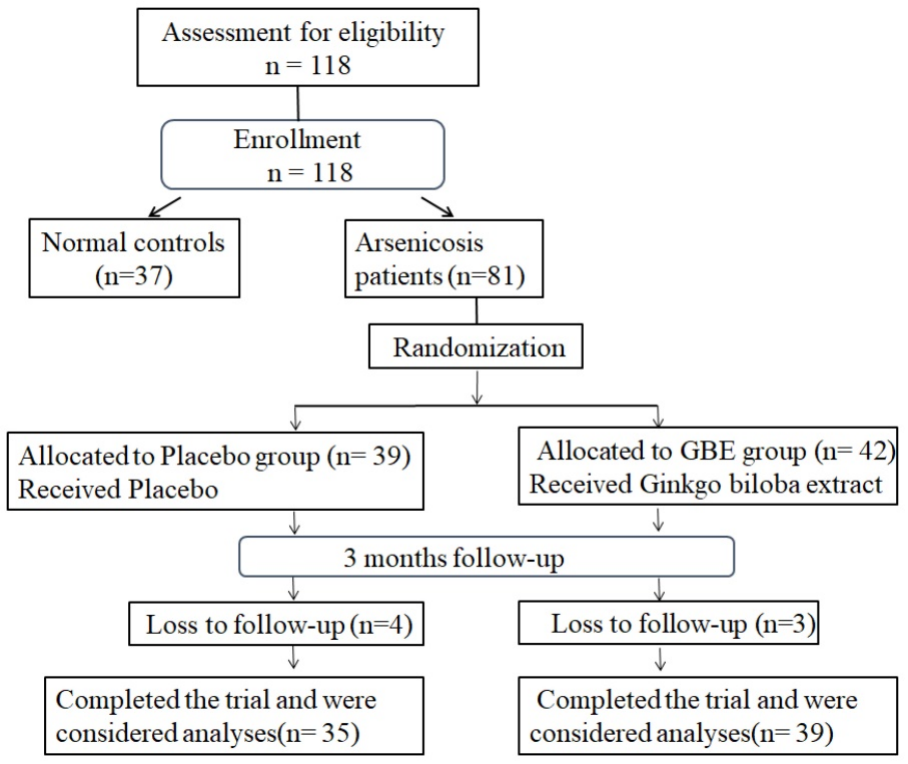
*** Study-flow diagram.***37 normal controls and 81 arsenicosis patients were investigated for the effects of arsenicosis on the balance of Th17/Treg cell in PBMCs and the levels of IL-17A, IL-6, and IL-10 levels in serum. The 81 arsenicosis patients were divided into two randomly allocated groups, with 39 in the placebo group and 42 in the GBE group. After 3 months, 35 (loss of 4 from 39) subjects in the placebo group and 39 (loss of 3 from 42) subjects in the GBE group were examined for the effects of GBE on the Th17/Treg cell balance of PBMCs and on the levels of IL-17A, IL-6, and IL-10 in serum.

For this study, 81 arsenicosis patients and 37 controls were enrolled. During 3 months of follow-up, seven arsenicosis patients were lost; 74 cases were completed and analyzed (35 cases in the placebo group and 39 cases in the GBE group) (Figure 1). The epidemiological characteristics of the study population are summarized in Table 1. There were no significant differences in terms of age, gender, smoking habits, or alcohol consumption between the arsenicosis patients and the control group (*P* > 0.05). Arsenic in hair, which may be derived from ingestion and/or from external contamination, has been used as a biomarker for arsenic body burden 31. The hair arsenic levels for the arsenicosis group were higher than those for the control group (0.23 vs 0.13 μg/g, *p* < 0.05) (Table 1).

### Arsenicosis disrupts the balance of Th17/Treg cell in PBMCs

**Figure 2:**
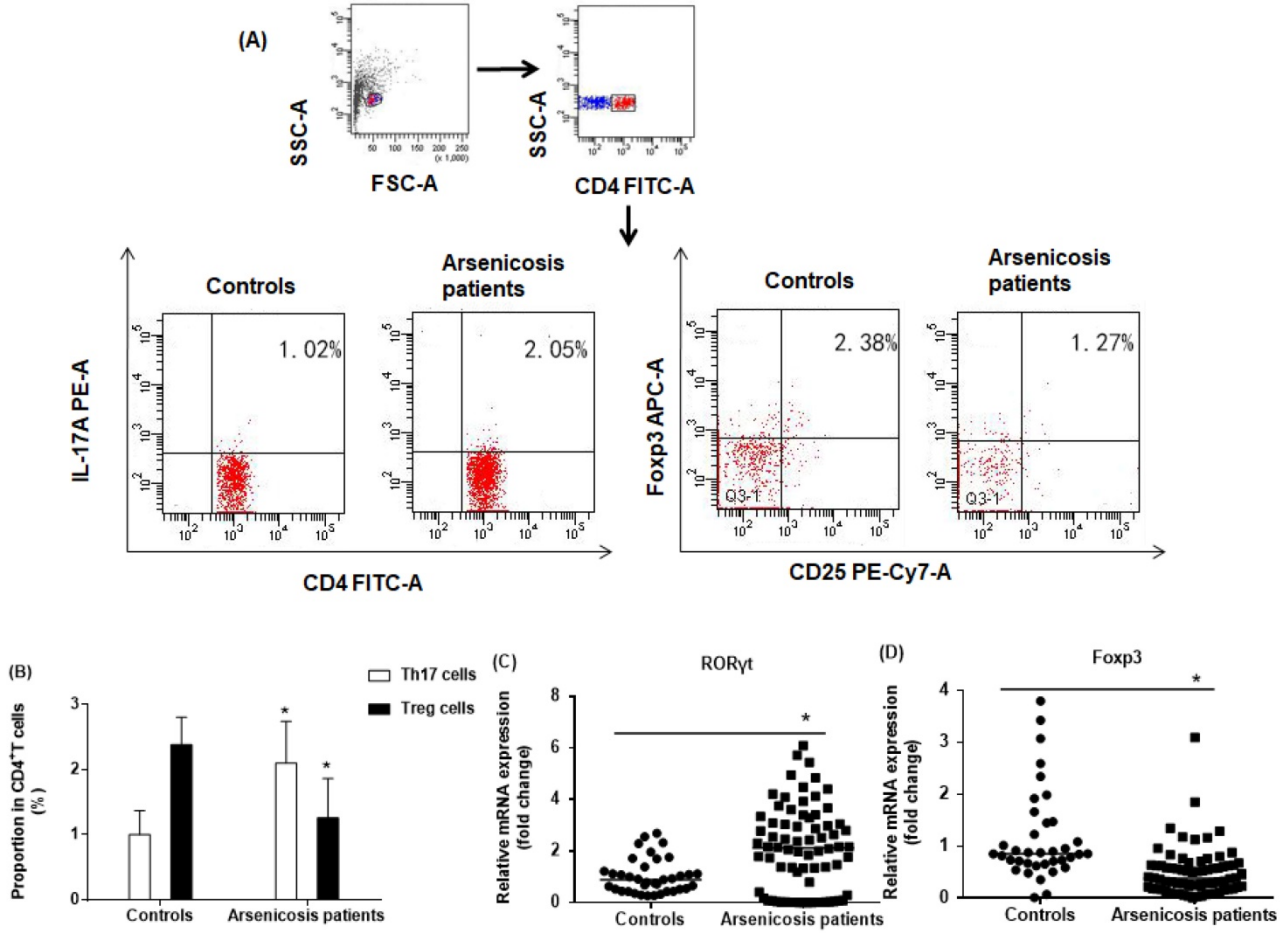
*** Arsenicosis disrupts the balance of Th17/Treg cell in PBMCs.***PBMCs: Peripheral blood mononuclear cells; Th17: T helper 17 cells; Treg: Regulatory T cell; RORγt: Retinoic acid-related orphan nuclear receptor γt; Foxp3: Forkhead/winged helix transcription factor 3. PBMCs were obtained from normal subjects (n=37) and from arsenicosis patients (n=81). (**A**) Representative flow cytometry (FCM) pictures of CD4^+^IL-17A^+^ Th17 cells and CD4^+^CD25^+^FoxP3^+^Treg cells in PBMCs of subjects. (**B**) The percentages of Th17 and Treg cells in PBMCs were measured by FCM. The mRNA levels of RORγt (**C**) and Foxp3 (**D**) were determined by qRT-PCR. Results are given as means ±SD. * *P* < 0.05, arsenicosis patients versus controls.

The proportions of CD4^+^CD25^+^FoxP3^+^ Treg and CD4^+^IL-17A^+^ Th17 cells in the total CD4^+^T cells of arsenicosis patients and controls were evaluated by flow cytometric analysis, which showed the prevalence of Treg and Th17 cells (Figure 2A). The numbers of Th17 cells were higher in PBMCs of arsenicosis patients (2.10 *±* 0*.*64 %) than in those of normal controls (1*.*00 *±* 0*.*37 %; *P <* 0*.*05). In contrast, the numbers of Treg cells were lower in the arsenicosis group (1.26 ± 0.60 %) compared to normal subjects (2.38 ± 0.42 %; *P <* 0*.*05) (Figure 2B). Thus, arsenicosis disrupts the Th17/Treg cell balance in PBMCs.

In Th17 cells, RORγt is a master transcription factor, and Foxp3 is a transcription factor for differentiation and function of Treg cells 12, 32. To confirm the imbalance in the ratio of Th17/Treg cells, we determined the relative mRNA levels of specific transcription factors in PBMCs of subjects in the two groups by qRT-PCR. As shown in Figure 2C, the mRNA levels of RORγt were higher in the arsenicosis group [2.11(0.15-3.14)] than in the control group [0.89(0.48-1.30); *p* < 0.05]. In contrast, the arsenicosis patients had lower mRNA levels of Foxp3 [0.38(0.18-0.63)] in their PBMCs than in those of the control group [0.86(0.66-1.46), *p* < 0.05] (Figure 2D). In sum, these results show that, in arsenicosis, there are abnormal expressions of RORγt and Foxp3, which may affect the balance of Th17/Treg cells.

### Arsenicosis causes increases of IL-17A and IL-6 levels and a decrease of IL-10 levels in serum

**Figure 3:**
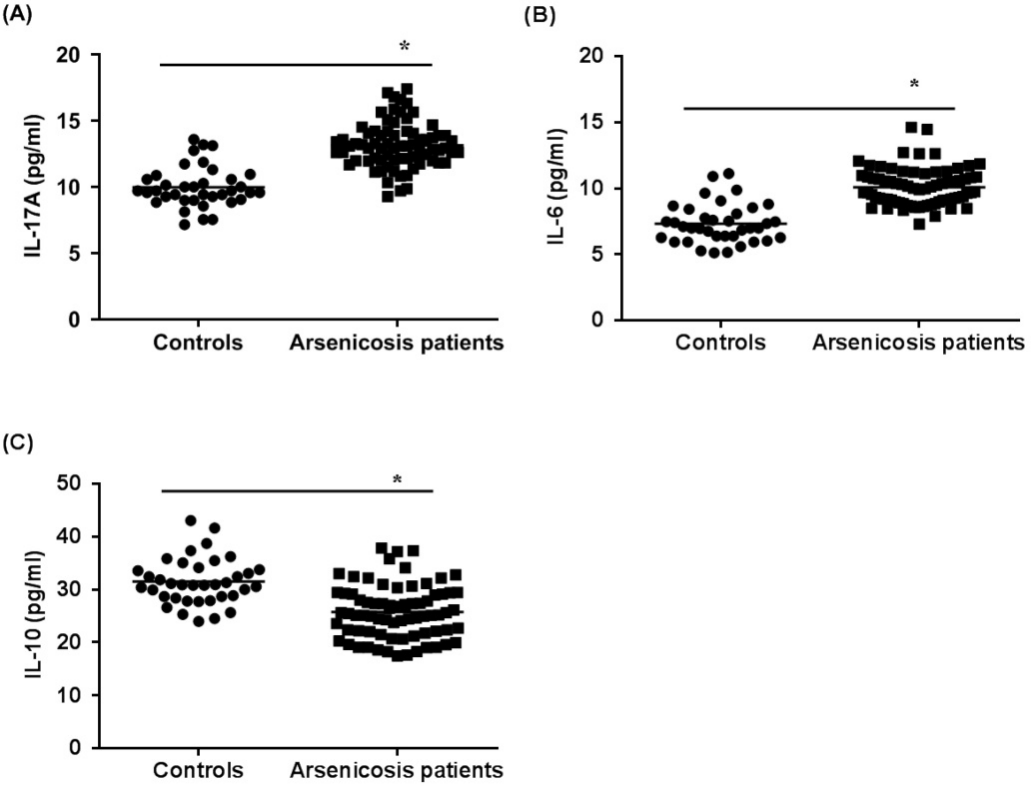
*** Arsenicosis causes increases of IL-17A and IL-6 levels and decreases of IL-10 levels in serum.***IL: interleukin. Serum were obtained from normal subjects (n=37) and arsenicosis patients (n=81). The levels of IL-17A (**A**), IL-6 (**B**), and IL-10 (**C**) in serum were measured using ELISA. Results are given as means ±SD. * *P* < 0.05, arsenicosis patients versus controls.

Next, we examined the cytokines related to Th17 cells and Treg cells in the sera of subjects by use of ELISA. As shown in Figure 3A and B, the levels of IL-17A and IL-6 in the arsenicosis group (IL-17A: 13.14 ± 1.71 pg/ml; IL-6: 10.09 ± 1.42 pg/ml) were higher than those in the control group (IL-17A: 9.98 ± 1.54 pg/ml; IL-6: 7.33 ± 1.49 pg/ml; both *p* < 0.05). Compared to that of the control group (31.50 ± 4.40 pg/ml), the levels of IL-10 in the arsenicosis group (25.77 ± 5.03 pg/ml) were lower; the differences were statistically significant (*p* < 0.05) (Figure 3C). Consequently, for arsenicosis patients, changes in the protein levels of cytokines related to Th17 and Treg cells were skewed toward Th17. Furthermore, we assessed whether the Th17 and Treg cells and their related cytokines were associated with the levels of arsenic exposure. These results showed that, for arsenicosis patients, the balance of Th17 and Treg cells was disrupted.

The levels of hair arsenic positively correlated with the percentage of Th17 cells (*r* = 0.403, *p* < 0.05) and negatively correlated with the percentage of Treg cells (*r* = - 0.350, *p* < 0.05). Further, there was a positive association of IL-17A and IL-6 expression (*r* = 0.525 and *r* = 0.427, both *p* < 0.05) and a negative correlation between hair arsenic and the expression of IL-10 (*r* = - 0.382, *p* < 0.05) (Table 2).

### GBE attenuates arsenicosis-induced disruption of the Th17/Treg cell balance in PBMCs

**Figure 4:**
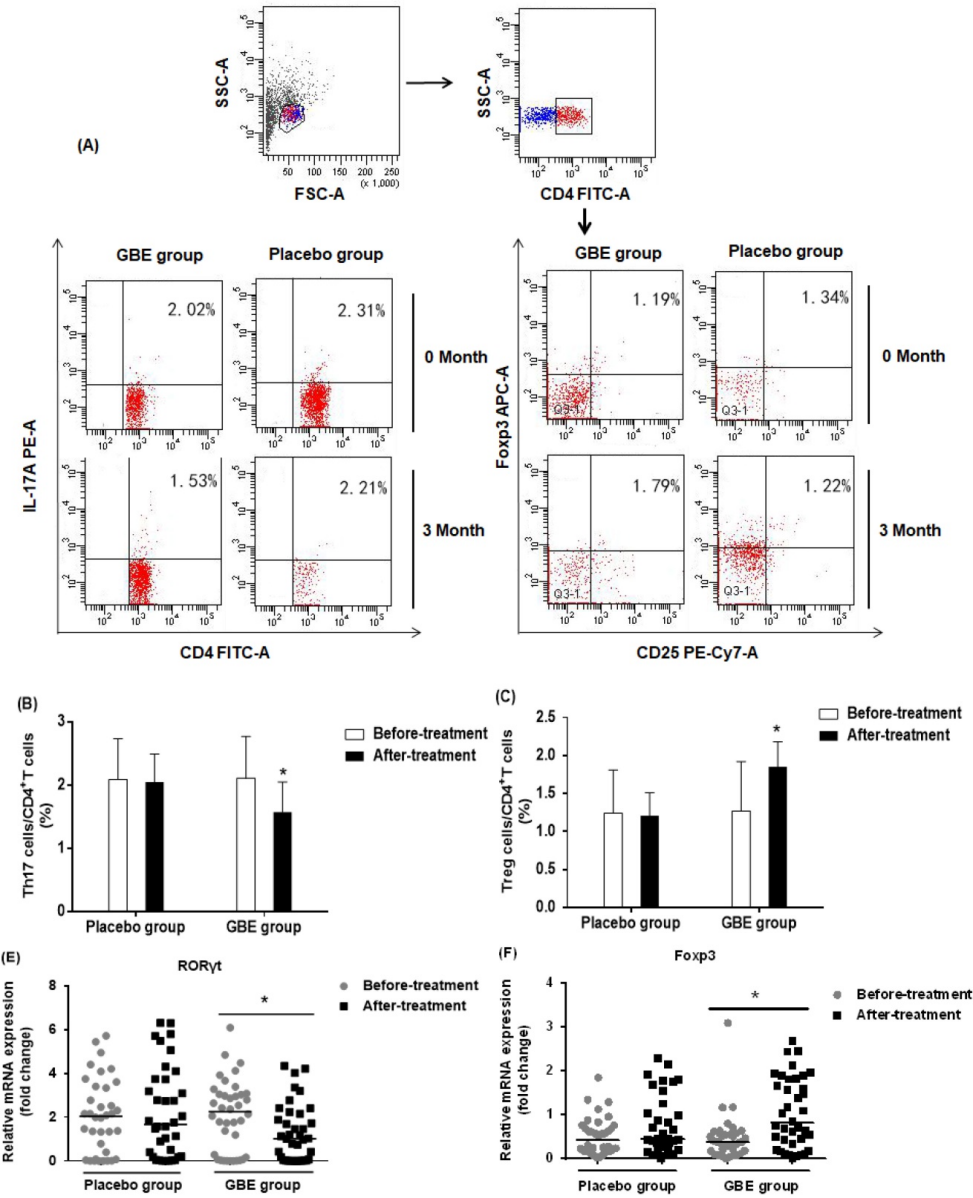
*** GBE reduces arsenicosis-induced disruption of the Th17/Treg cell balance in PBMCs.***After arsenicosis patients were administered placebo (group, n=35) or GBE (group, n=39) for 3 months, PBMCs were obtained as described in Methods. (**A**) Representative FCM pictures of CD4^+^IL-17A^+^ Th17 cells and CD4^+^CD25^+^FoxP3^+^Treg cells in the two groups. The percentage of Th17 cells (**B**) and Treg cells (**C**) in PBMCs were measured by FCM. The mRNA levels of RORγt (**D**) and Foxp3 (**E**) were measured by qRT-PCR. Results are given as means ±SD. * *P* <0.05 versus before treatment.

*Gingko biloba* has been used to enhance immune function in arsenic-poisoned rats and to relieve kidney damage 29. We followed up 74 arsenicosis patients who received a course of 3-month placebo or GBE therapy. For all arsenicosis patients (n =39), the numbers of CD4^+^CD25^+^FoxP3^+^Treg and CD4^+^IL-17A^+^Th17 cells were affected following GBE treatment (Figure 4A). For the treated patients, GBE reduced the numbers of Th17 cells from 2.11 ± 0.66 % to 1.57 ± 0.48 % and increased Treg cell numbers from 1.27 ± 0.65 % to 1.85 ± 0.33 % (Figure 4B, C). In contrast, in the peripheral blood of arsenicosis patients after 3 months of treatment with placebo, there was no significant difference in numbers of Th17 and Treg cells.

To assess the mechanism underlying the protective effect of GBE against an imbalance in the ratio of Th17/Treg cells, we determined the mRNA levels of RORγt and Foxp3 in PBMCs of arsenicosis patients before and after therapy. At the end of the study, the expression of RORγt was lower in the GBE group compared with baseline values (*P <* 0.05). Foxp3 mRNA levels were higher following GBE treatment, but this variable showed no significant difference in the placebo group (*P* > 0.05) (Figure 4D, E). These findings indicated that, in PBMCs, GBE attenuated the arsenicosis-induced disruption of the Th17/Treg cell balance, which could be associated with higher expression of Foxp3 and lower expression of RORγt.

### GBE prevents the increases of IL-17A and IL-6 levels and the decrease of IL-10 levels in serum of arsenicosis patients

**Figure 5:**
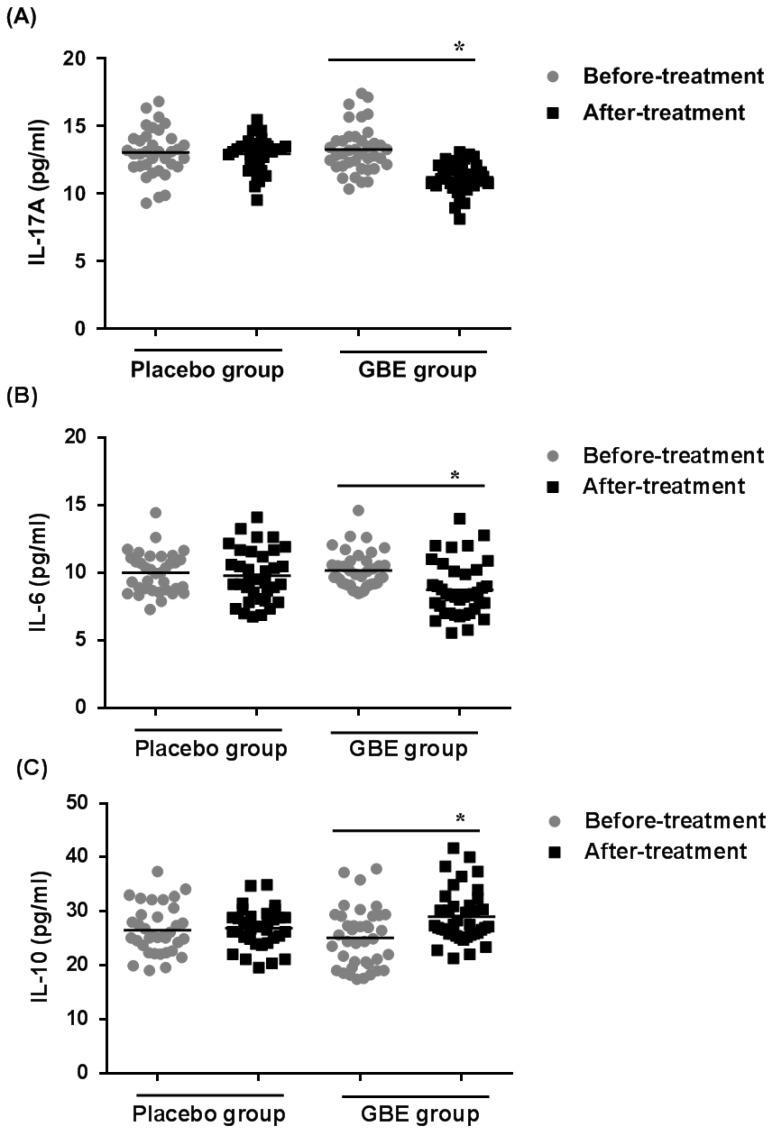
** GBE prevents increases of IL-17A and IL-6 levels and decreases of IL-10 levels in serum of arsenicosis patients.** After arsenicosis patients were administered placebo (group, n=35) or GBE (group, n=39) for 3 months, sera were obtained as described in Methods. The levels of IL-17A (**A**), IL-6 (**B**), and IL-10 (**C**) in serum were measured using ELISA. Results are given as means ±SD; * *P* <0.05 versus before treatment.

To determine if secretion of cytokines by Th17 and Treg cells was altered in arsenicosis patients treated with GBE, the levels of IL-17A, IL-6, and IL-10 cytokines were examined in the sera of patients in the treatment and placebo groups. After therapy (3 months), the GBE group showed reductions in IL-17A levels and IL-6 levels (both *P <* 0.05), indicating that GBE had an anti-inflammatory effect (Figure 5A, B). Before treatment, the serum levels of IL-10 were lower in the GBE group, but they were restored to normal after treatment. For the placebo group, there were no significant differences in cytokine levels after 3 months (Figure 5C). These results revealed that arsenicosis induces increases of IL-17A and IL-6 levels and a decrease of IL-10 levels in serum, which is prevented by GBE.

## Discussion

Our previous research describes skin lesions and other damage caused by arsenic poisoning 30. In the present investigation, we explored the mechanism of arsenic exposure in the damage to skin, liver, and other tissues through influence on the Th17/Treg cell balance. In the arsenicosis group were 81 villagers who were evaluated between March 2017 and June 2017 according to the Standard of Diagnosis for Endemic Arsenism (WS/T 211-2015, Ministry of Health of the People's Republic of China). Since hair arsenic levels reflect arsenic absorption related to arsenicosis 33, 34, it is practical to use hair arsenic concentrations as a biomarker of exposure for arsenicosis. In the present investigation, our data showed that arsenic levels in hair of the arsenicosis group (median, 0.23, and 5-95%, 0.10-0.57) were higher than those of controls (median, 0.13, and 5-95% 0.04-0.46), which indicated that arsenic in hair is a biomarker of arsenicosis.

Th17 cells, which were recently found after the discovery of a new type of inflammatory cytokine, IL-17A, are involved in host defense against a variety of pathogens as well as in the pathogenesis of various inflammatory conditions 44, 45. Treg cells have suppressor activity and function in the maintenance of self-tolerance, which, when diminished, contributes to development of diseases 46. The present data showed that patients with arsenicosis exhibited an increase in peripheral Th17 numbers and levels of Th17-related cytokines (IL-17A and IL-6), as well as lower Treg numbers and Treg-related cytokines (IL-10) compared to the control group. This result is in contrast to previous reports of investigations with experimental animals 16, 17. The variations may be related to different types of exposure, doses, and/or experimental systems. The present results indicated that a Th17/Treg imbalance existed in arsenicosis patients, consistent with results demonstrated by Hernández-Castro et al 19, showing that the Th17/Treg ratios correlated with the accumulated arsenic load.

The differentiation processes for Th17 and Treg cells are related. In naïve CD4^+^ T cells, TGF-β induces the expression of both Foxp3 and RORγt, but the former is recessive and promotes RORγt in the presence of IL-6, shifting the balance from Treg to Th17 cells in inflammatory conditions 47. Overexpression of the pro-inflammatory interleukins, IL-6 and IL-8, is associated with arsenic-induced malignant transformation of human bronchial epithelial cells and urothelial cells 48, 49. Individuals exposed to arsenic through drinking water have elevated levels of plasma pro-inflammatory mediators, including tumor necrosis factor-α (TNF-α), IL-6, IL-8, and IL-12, as well as lower plasma levels of the anti-inflammatory cytokine, IL-10 50, 51. Consistent with these observations, our results showed that the expressions of IL-6 and IL-17A, both of which promote the differentiation of Th17 cells, were high in patients with arsenicosis caused by burning coal, and they positively correlated with hair arsenic levels. These results demonstrated that chronic exposure to arsenic induced inflammatory responses and elevated pro-inflammatory cytokines such as IL-6 and IL-17A, leading to Th17 cell differentiation, development, and maintenance.

In contrast, Treg cells suppress inflammation and immune responses through production of IL-10, a cytokine with immunosuppressive properties and anti-inflammatory activity 52. The present results showed that IL-10 levels for the arsenicosis group were lower than those for the control group, and, combined with the results for Treg cells, suggest that when arsenic exposure is excessive, the capacity of Treg cells and IL-10 in inhibiting the inflammatory response may not be sufficient to complete the anti-inflammatory function, thus leading to an enhanced inflammatory response in arsenicosis patients. A high Th17/Treg ratio may result in the loss of tolerance and regulation, and ultimately to a sustained, chronic inflammatory response, which is a characteristic of autoimmune, inflammatory, and malignant diseases 53, 54. This suggests that, for arsenicosis patients, arsenic causes a Th17/Treg functional imbalance, which leads to less immune tolerance and to a disorder in the regulation of inflammatory responses, thus facilitating the occurrence and development of immune injuries induced by arsenic. However, the mechanism underlying these changes is not well understood.

RORγt, a transcription factor, is involved in the development and function of Th17 cells 12. Foxp3 is a master gene responsible for the immune-suppressing activity of Treg cells 32. To understand the effects of arsenic exposure on transcription factors, we assessed the alteration of Foxp3 and RORγt expressions in patients with arsenic poisoning. Arsenic exposure was associated with greater RORγt expression and lower Foxp3 expression. These results demonstrated an imbalance in the ratio of Th17/Treg cells at the transcription factor level and a Th17 cell-based pattern in patients exposed to arsenic by burning coal in unventilated indoor stoves.

GBE affects immune responses by increasing phagocytosis of macrophages; increasing production of anti-SRBC antibodies; and reducing the expressions of IL-8, TNF-α, and IL-1^55^, ^56^. We showed that GBE ameliorates the unbalanced Th17/Treg response in the blood of arsenicosis patients. Following GBE treatment, there were lower numbers of Th17 cells and higher numbers of Treg cells. Furthermore, the ratio of Th17/Treg cells was lower at the end of treatment compared with the baseline. Our results indicated a role of Th17/Treg imbalance in the pathogenesis of arsenic poisoning. For patients with schizophrenia, GBE increases the numbers of T lymphocytes and adjusts the balance of peripheral T lymphocyte subsets, thereby enhancing immune function 57. Together, these data imply that GBE influences the Treg/Th17-mediated response and suggest a beneficial effect of using GBE in the treatment of arsenicosis.

GBE exhibits anti-inflammatory effects by inhibiting pro-inflammatory mediators and signaling pathways 58, 59. It inhibits production of the pro-inflammatory cytokines, IL-1β and TNF-α, but up-regulates production of the anti-inflammatory cytokine, IL-10, and its receptor IL-10R 60, 61. Consistent with these findings, our results showed that administration of GBE decreased the expression of the serum inflammatory cytokines, IL-17A and IL-6, but increased expression of IL-10, compared with the baseline. The changes of cytokine expression are consistent with the changes in Th subsets. These results implied that, for arsenicosis patients, GBE suppressed the inflammatory response. In a pro-inflammatory environment, Treg cells release IL-17A; in the absence of inflammation, TGF-β promotes Treg differentiation to maintain immune tolerance 62, 63. Thus, in considering the effect of *Gingko biloba* treatment on the immune suppression and kidney damage in arsenic poisoned rats 29 and the results of this study, we conclude that GBE has a role in the balance of the Th17/Treg axis and shifts this balance to produce an anti-inflammatory effect. For arsenicosis patients, their inflammatory imbalance can be modified to restore immune homeostasis, thereby improving their immune function.

To characterize the molecular mechanisms underlying the immunoregulatory effect of GBE, we examined the transcription factors, Foxp3 and RORγt, which are specific, respectively, for Treg and Th17 cells. GBE treatment decreased the expression levels of RORγt but increased expression of Foxp3 in peripheral blood, supporting the concept that, for patients with arsenicosis, GBE restores a balanced Th17/Treg response via regulating the levels of RORγt and Foxp3. The JAK-mediated phosphorylation of STAT3 regulates the expression of RORγt; a STAT3 deficiency in CD4^+^T cells results in impaired Th17 development and a deficiency in RORγt 64. In addition, STAT3 is an inhibitor of Foxp3 65. Ginkgolide, a compound present in GBE, reduces the low-grade vascular inflammation caused by high glucose levels through regulation of a STAT3-mediated pathway 66. Whether GBE regulates the expression of RORγt and Foxp3 in arsenicosis patients through this mechanism, and thereby improves the immune status of the body, remains to be studied.

## Materials and Methods

### Study population

The investigation site was Changqing village, Xingren County, Guizhou Province, China, where the residents use arsenic-containing coal for cooking and are thus exposed to arsenic via polluted food and air. According to the Standard of Diagnosis for Endemic Arsenism (WS/T 211-2015, Ministry of Health of the People's Republic of China), 81 villagers were designated as the arsenicosis group between March 2017 and June 2017. The others (37), who did not use coal containing high concentrations of arsenic and exhibited no signs of arseniasis, were designated as the control group (Table 1). All participants were permanent residents of the local area and were matched for age and sex. Exclusion criteria included a history of occupational exposure; a history of hypertension, immunodeficiency, or autoimmune-related disease; a one-month history of infection; and a recent history of consuming seafood or drugs that could affect immune function or the excretion of arsenic. This study was approved by the Ethical Committee of Nanjing Medical University (No 2017-534), and written informed consents were obtained from all individuals.

### Study design

The study was designed as a randomized, double-blind, placebo-controlled trial. Arsenicosis patients were divided into two randomly allocated groups (placebo or GBE) by random permuted blocks within the strata (age and sex) method. Patients in the GBE group were subjected to therapy that consisted of 3 months of oral administration of GBE [3 tablets/day, each tablet containing flavonoid glycosides (19.2 mg) and terpenoids (4.8 mg), manufactured by Guizhou Xinbang Pharmaceutical Co. Ltd]. The placebo group received three placebo tablets (starch) per day. The doses were maintained at those levels until the end of the trial. To ensure compliance and reliability of ratings across the study, all patients were controlled through phone calls every week and treated by full-time medical staff. All subjects and investigators were blind to treatment conditions and remained blinded until after data analysis. Only a staff member at Standard Process knew the assignment. At baseline and at the final follow-up at month 3, physical examinations were performed by medical professionals who worked in the Forty-fourth Hospital of the Chinese People's Liberation Army.

### Interviews and sample collection

A structured questionnaire was used to record demographic data about participants, including gender, age, lifestyle, and arsenic exposure. After receiving informed consent, fasting venous blood samples were collected at baseline and after intervention. Samples of blood were collected into tubes containing 0.2 mL of sodium heparin and in non-anticoagulant tubes. PBMCs were isolated from heparinized blood by Ficoll density gradients for flow cytometric analysis and extraction of total RNA. Sera were obtained after centrifugation of blood samples and stored at -80 ℃ for the measurement of cytokine concentrations. To measure the arsenic content of hair of the subjects, samples were collected within 3 cm of the hair root and stored in plastic zipper bags.

### Arsenic concentrations in hair measured by ICP-MS

Hair samples were analyzed within one month after collection. Details of the analytical method have been described 67. The hair samples were washed with high-purity deionized water, soaked in acetone, dehydrated with ether, dried in an oven at 60 °C, and cut into pieces 0.5-cm long. After that, the hair samples were digested for 1 h with 6 mL concentrated HNO_3_ using a microwave digestion instrument (Anton Paar, Multiwave GO, Sweden). Total arsenic concentrations in hair samples were determined by hydride generation, inductively coupled plasma mass spectrometry (ICP-MS) (Thermo Fisher, XSeries2, USA).

### Flow cytometric analysis of Th17 and Treg cells

Isolated PBMCs were suspended at a density of 2×10^6^ cells/ml in RPMI-1640 medium (Hyclone, USA) supplemented with 10% fetal bovine serum (Gibco, USA). For Th17 analysis, cell suspensions were transferred to wells of 24-well plates. Cultures were incubated with 4 μL of Leukocyte Activation Cocktail with BD GolgiPlug™ (BD Biosciences, USA) for 5 h at 37 ℃ in a 5% CO_2_ humidified incubator. The cells were then incubated with fluorescein isothiocyanate (FITC) antihuman CD4 (BD Biosciences, USA) at 4 ℃ for 30 min. For Treg analysis, cells were labeled with FITC antihuman CD4 (BD Biosciences, USA) and PE-conjugated anti-human CD25 (BD Biosciences, USA) at 4 ℃ for 30 min. Following surface staining, cells were washed in PBS and fixed with fixation buffer (BD Biosciences, USA). Afterwards, the cells were washed twice with 1× permeabilization buffer (BD Biosciences, USA) and then stained with phycoerythrin antihuman IL-17A (BD Biosciences, USA) for detection of Th17 or with APC anti-human Foxp3 (BD Biosciences, USA) for detection of Treg cells. Isotype controls (BD Biosciences, USA) were treated to allow compensation and to confirm antibody specificity. The stained cells were analyzed with a FACSCalibur flow cytometer (BD Biosciences). Data analysis was accomplished with CellQuest Pro software (BD Biosciences).

### Total RNA isolation and quantitative real-time reverse transcription PCR

Total RNA was isolated from PBMCs by using Trizol reagent (Thermo Fisher/Invitrogen, USA). RNA was then converted to cDNA with Revert Aid TM first-strand cDNA synthesis Kits (Thermo Fisher, USA) according to manufacturer's instructions. Quantitative real-time reverse transcription PCR (qRT-PCR) was performed with a CFX96 Real-Time PCR Detection System (Bio-Rad, California) by incubating the cDNA with primers and Power SYBR Green Master Mix (TaKaRa, Japan). Primers for Foxp3 (forward: *5′-TGGAGGAACTCTGGGAATGTG-3′*, reverse: *5′-AGGCTTCATCTGTGGCATCAT-3′*); RORγt (forward: *5′-CTGCTGAGAAGGACAGGGAG-3′*, reverse: *5′-AGTTCTGCTGACGGGTGC- 3′*); and glyceraldehyde 3-phosphate dehydrogenase (GAPDH) (forward: *5′-GGACCTGACCTGCCGTCTAG-3′*, reverse: *5′-GTAGCCCAGGATGCCCTTGA-3′*) were synthesized by GENEray Biotechnology Co. Ltd (Shanghai, China). Relative gene expression was calculated by using the comparative CT method. GAPDH was used as a housekeeping gene for normalization.

### Measurement of Serum Cytokines

The serum levels of IL-17A, IL-6, and IL-10 were measured by enzyme-linked immunosorbent assay (ELISA), following the manufacturer's instructions (ELISA kits, all from BD Biosciences). The concentrations were calculated from a standard curve according to the manufacturer's protocol and read on a Max200 instrument (Bio-Tek, California). All samples were measured in duplicate.

### Statistical analysis

Statistical analysis was performed using SPSS PC Statistics (version 13.0; SPSS Inc., Chicago, IL, USA). For quantitative data, after testing for normality and variance homogeneity, the independent sample test and paired t test were used for comparison between before and after variables and within groups, respectively. Data for relative expression of transcription factors were described as median (interquartile range), and cell numbers and cytokine expression were expressed as the means ± SD. A two-tailed Chi-square test was used to compare the categorical variables of current smokers, alcohol use, and sex between the arsenicosis group and the control group. Differences of hair arsenic levels between groups were analyzed by the Mann-Whitney Test. A Spearman's rank correlation coefficients analysis was applied to analyze the association between two variables. Values of the expressions of Foxp3 and RORγt were in-transformed to fit the corresponding models. Differences were considered statistically significant when *p* < 0.05.

## Conclusions

**Figure 6:**
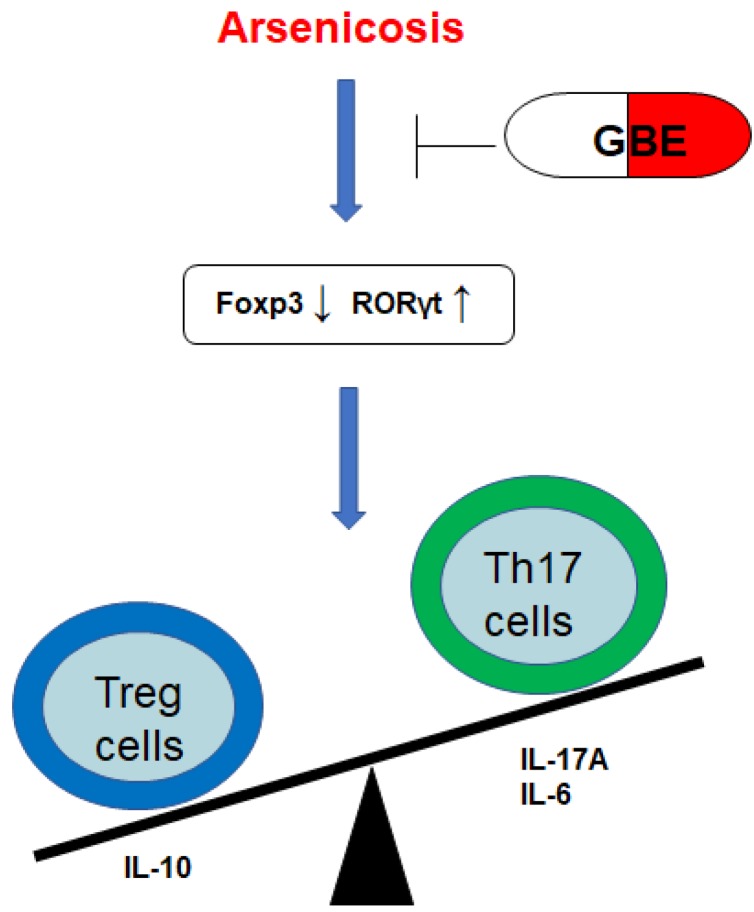
*** A schematic diagram of how GEB attenuates the arsenicosis-induced imbalance of Th17/Treg cells.***Arsenicosis causes increases of the transcription factor RORγt, which elevates the levels of Th17 cells and Th17-related cytokines (IL-17A and IL-6). However, arsenicosis induces decreases of the transcription factor Foxp3, which decreases the levels of Treg cells and a Treg-related cytokine (IL-10). GEB attenuates the arsenicosis-induced imbalance of Th17/Treg cells, which indicates that the imbalance of Th17/Treg cells may be involved in the pathogenesis of arsenicosis and that GEB may be involved in the treatment of arsenicosis through influencing the Th17/Treg balance.

In summary, arsenicosis causes increases of the transcription factor RORγt, which elevates the levels of Th17 cells and Th17-related cytokines (IL-17A and IL-6). However, arsenicosis induces decreases of the transcription factor Foxp3, which decreases the levels of Treg cells and a Treg-related cytokine (IL-10). GEB attenuates the arsenicosis-induced the imbalance of Th17/Treg cells, which indicates that the imbalance of Th17/Treg cells may be involved in the pathogenesis of arsenicosis and that GEB may facilitate the treatment of arsenicosis through accentuating the influence on the Th17/Treg balance (Figure 6). Therefore, our data offer evidence for a skewed balance of Th17/Treg pro- and anti-inflammatory T cell subsets in peripheral blood of arsenicosis patients. In addition, we show that administration of GBE to patients with arsenism mediates the immunity balance by suppressing the pro-inflammatory Th17 cells and related cytokines and by up-regulating Treg and anti-inflammation cytokines. Thus, our results suggest that an imbalance of the ratio of Th17/Treg cells is involved in the pathogenesis of arsenicosis and that, for patients with arsenic poisoning, GBE treatment provides favorable immunological effects.

### Author Contributions

Conceptualization, Shiqing Xia, Qian Sun, and Zhonglan Zou; Data curation, Yonglian Liu and Xiaolin Fang; Formal analysis, Baofei Sun and Shaofeng Wei; Funding acquisition, Aihua Zhang and Qizhan Liu; Investigation, Shiqing Xia, Qian Sun, and Zhonglan Zou; Methodology, Yonglian Liu and Xiaolin Fang; Formal analysis, Baofei Sun and Shaofeng Wei; Project administration, Aihua Zhang; Resources, Aihua Zhang; Supervision, Aihua Zhang and Qizhan Liu; Writing-original draft, Shiqing Xia and Qian Sun; Writing-review and editing, Aihua Zhang and Qizhan Liu.

## Figures and Tables

**Table 1:** Baseline characteristics of the study population.

Variable	Arsenicosis group(n=74)	Controls(n=37)	*P* Value
Age (years, mean ±SD)	50.47±6.85	48.03±8.24	0.125^a^
Gender, n (%)			
Male	46(62.2%)	24(64.9%)	0.781^b^
Female	28(37.8%)	13(35.1%)	
Current smokers, n (%)			
Yes	30(40.5%)	12(32.4%)	0.576^b^
No	44(59.5%)	25(67.6%)	
Alcohol use, n (%)			
Yes	21(28.4%)	13(35.1%)	0.467^b^
No	53(71.6%)	24(64.9%)	
Arsenic exposure (median, 5-95%)			
Hair arsenic (μg/g hair)	0.23(0.10-0.57)	0.13(0.04-0.46)	< 0.001^c^

^a^ Independent-sample t-test. ^b^ Two-tailed χ^2^ test. ^c^ Mann-Whitney Test. Values in bold are statistically significant (P < 0.05).

**Table 2:** Relationship between arsenic levels and percentage of Th17 cells, Treg cells, and related cytokines.

Variable	Hair arsenic	
	*r*	*p*
Th17 cells/CD4^+^T cells (%)	0.403	<0.001
Treg cells/CD4^+^T cells (%)	-0.350	0.002
IL-17A (pg/ml)	0.525	<0.001
IL-6 (pg/ml)	0.427	<0.001
IL-10 (pg/ml)	-0.382	<0.001

## Abbreviations

GB: *Ginkgo biloba*
GBE: *Ginkgo biloba* extract
PBMCs: Peripheral blood mononuclear cells
Th: T-helper
Th17: T helper 17 cell
Treg: Regulatory T cell
TNF-α: Tumor necrosis factor-α
RORγt: Retinoic acid-related orphan nuclear receptor γt
Foxp3: Forkhead/winged helix transcription factor 3
IL: Interleukin
IL-17A: Interleukin 17A
IL-6: Interleukin 6
IL-10: Interleukin 10
TGF-β1: Transforming growth factor-β1
JAK: Janus kinase
STAT3: Signal transducers and activators of transcription 3
ICP-MS: Inductively coupled plasma mass spectrometry
qRT-PCR: Quantitative real-time reverse transcription PCR
ELISA: Enzyme-linked immunosorbent assay
